# Identification of Aryl Polyamines Derivatives as Anti-*Trypanosoma cruzi* Agents Targeting Iron Superoxide Dismutase †

**DOI:** 10.3390/pharmaceutics15010140

**Published:** 2022-12-31

**Authors:** Rubén Martín-Escolano, Daniel Molina-Carreño, Javier Martín-Escolano, Mª Paz Clares, Cristina Galiana-Roselló, Jorge González-García, Nuria Cirauqui, José M. Llinares, María José Rosales, Enrique García-España, Clotilde Marín

**Affiliations:** 1 Laboratory of Molecular & Evolutionary Parasitology, RAPID Group, School of Biosciences, University of Kent, Canterbury CT2 7NJ, UK; 2 Department of Parasitology, University of Granada, Severo Ochoa s/n, 18071 Granada, Spain; 3 Unit of Infectious Diseases, Microbiology and Preventive Medicine, Institute of Biomedicine of Seville (IBiS), University Hospital Virgen del Rocío/CSIC/University of Seville, 41013 Seville, Spain; 4 Instituto de Ciencia Molecular (ICMol), Departamento de Química Inorgánica, Universidad de Valencia, 46980 Paterna, Spain; 5 Departamento de Farmacia, Facultad de Ciencias de la Salud, Universidad CEU Cardenal Herrera, C/Ramón y Cajal, s/n, 46115 Alfara del Patriarca, Spain; 6 EMBL Grenoble, 71 Avenue des Martyrs, 38000 Grenoble, France; 7 Departamento de Química Orgánica, Universidad de Valencia, C/Dr. Moliner s/n, 46100 Burjassot, Spain

**Keywords:** chagas disease, chemotherapy, drug discovery, aryl polyamines, *Trypanosoma cruzi*

## Abstract

Chagas disease (CD) is a tropical and potentially fatal infection caused by *Trypanosoma cruzi*. Although CD was limited to Latin America as a silent disease, CD has become widespread as a result of globalization. Currently, 6–8 million people are infected worldwide, and no effective treatment is available. Here, we identify new effective agents against *T. cruzi*. In short, 16 aryl polyamines were screened in vitro against different *T. cruzi* strains, and lead compounds were evaluated in vivo after oral administration in both the acute and chronic infections. The mode of action was also evaluated at the energetic level, and its high activity profile could be ascribed to a mitochondria-dependent bioenergetic collapse and redox stress by inhibition of the Fe-SOD enzyme. We present compound **15** as a potential compound that provides a step forward for the development of new agents to combat CD.

## 1. Introduction

Chagas disease (CD) is a potentially fatal tropical infection naturally caused by the insect-transmitted parasite *Trypanosoma cruzi*, although the oral route involving parasite-contaminated food, blood transfusion, organ transplantation, congenital transmission and laboratory accidents are other important routes of transmission. Although CD was limited for many decades to Latin America as a silent and silenced disease [1], CD has become widespread as a result of globalization, with recorded outbreaks in North America, Japan, Europe and Oceania [2,3,4,5]. Currently, 6–8 million people are infected, 12–14 thousand people die annually, and 70–100 million people are hypothesized to be at risk of infection [5,6,7].

The World Health Organization (WHO) classifies CD as the most important parasitic disease and the most prevalent of the poverty-caused and poverty-promoting neglected tropical disease (NTD) in Latin America, and the Drug for Neglected Diseases *Initiative* (DNDi) declares CD a major public health problem worldwide. However, 110 years after its discovery there is no effective treatment or vaccine and much remains to be done to address the challenge properly [8]. Gaps in the knowledge of *T. cruzi* biology and the complex pathology of CD have been major factors in limiting progress [5]. The front-line treatments are limited to two obsolete nitroheterocyclic drugs discovered approximately 50 years ago—benznidazole (BZN) and nifurtimox—and lead to serious drawbacks. They require prolonged treatment, present toxic side effects, and their effectiveness gets reduced the longer the individual has been infected [3,9,10]. Besides these well-known limitations, other drawbacks are the natural resistance of the *T. cruzi* variants to the drugs used [11] and the cross-resistance of current drugs as they are activated within the parasite by the same mitochondrial type 1 nitroreductase [11,12].

The goal is to eradicate the parasite from infected individuals, thereby decreasing the likelihood of both symptomatic CD and the spread of the disease. In brief, there is an urgent need for effective, safe, tolerable and accessible new drugs against CD. Here, we identify aryl polyamines agents against *T. cruzi*. These aryl polyamines were selected in view of previous work in which some of us showed the ability of related macrocyclic polyamines to inhibit superoxide dismutase enzymes of the parasite [13]. Moreover, as some compounds had potential anticancer activity due to their ability to sequester Zn(II), it resulted interesting to check also their antiparasitic properties in relation to this point [14]. The strategy applied in this early drug discovery pipeline (DDP) was based on the target product profile (TPP) and the criteria stablished by different authors for CD [8,15,16,17,18,19]. In vitro screenings were carried out against different *T. cruzi* strains (including a BZN-resistant strain), and in vivo evaluations were performed after oral administration in both the acute and chronic infections in mouse model. Compound **15** meets the most stringent in vitro requirements and exhibits a better in vivo profile than BZN as a potential anti-*T. cruzi* agent. Finally, tests were conducted at the glycolytic and mitochondrial levels to elucidate its mode of action (MoA).

## 2. Materials and Methods

### 2.1. Chemistry

All the reagents were obtained by commercial suppliers (Sigma-Aldrich) and were used without further purification. To obtain hydrochloride salts, it was used dry ethanol from secure sealed bottles (Seccosolv, max. 0.01% H_2_O). Thin-layer chromatography (TLC) was carried out on aluminium oxide or silica gel precoated plates (60 PF254, Merck). The detection of the compounds was achieved by fluorescence quenching at 254 nm or staining with ninhydrin (0.3% ethanolic solution). Column chromatography purifications were performed on neutral aluminum oxide or silica gel 60 (0.040–0.063 mm) from Merck. NMR spectra were recorded at room temperature on a Bruker Advance DPX300 spectrometer operating at 299.95 MHz for ^1^H and at 75.43 MHz for ^13^C and referenced to tetramethylsilane or 3-(trimethylsilyl) propionic-2,2,3,3-d_4_ acid sodium salt. Chemical shifts (δ values) and coupling constants (*J* values) are given in ppm and Hz, respectively. High-resolution mass spectrometry (HRMS) was performed with electron spray ionization (ESI) recorded on an Esquire 300 (Bruker) by electrospray positive mode (ES+) or negative mode (ES–).

General synthesis of compounds **1**–**13**. The synthesis of the starting material diphthalimido-3-azapentane was carried out as described in [20]. The precursor 4-(2-naphthylmethyl)-1,4,7-triazaheptane was synthesised following the general procedure described in [21]. The corresponding carboxaldehyde (2.1 equiv) dissolved in EtOH (100 mL) was added dropwise to a solution of 4-(2-naphthylmethyl)-1,4,7-triazaheptane (1 equiv) in EtOH (50 mL). The reaction mixture was stirred at room temperature under nitrogen atmosphere for 4 h. NaBH_4_ (10 equiv.) was then added portion wise. The mixture was stirred for 6–12 h, and the solvent was then vacuum-evaporated to dryness. The residue was treated with water (100 mL) and extracted with CH_2_Cl_2_ (3 × 50 mL). The organic phase was washed with brine, dried with anhydrous Na_2_SO_4_, and filtered, and the solvent was evaporated to dryness. The residue was purified by column chromatography on silica gel or aluminium oxide to give an oil. The resulting oil was dissolved in the minimum of dry EtOH or CH_2_Cl_2_, and the amine was precipitated as its hydrochloride salt with 4 M HCl/dioxane solution (3 mL).

The synthesis and characterization of **2**, **6**, **8** and **9**–**13** have been described in [14], **1** has been reported on [22], **3** has been described in [21], **7** has been presented in [23], **14** has been reported on [24], and **15**–**16** have been described in [25].

*1,7-Bis(4-quinolylmethyl)-4-(2-naphthylmethyl)-1,4,7-triazaheptane Pentahydrochloride* (**4**). 4-(2-naphthylmethyl)-1,4,7-triazaheptane (0.54 g, 2.22 mmol) was reacted with 4-quinoline carboxaldehyde (0.73 g, 4.66 mmol) according to the general procedure described above. The residue was purified by column chromatography on neutral aluminium oxide (CHCl_3_/MeOH 70:30) to give a brown oil, which then precipitated as its pentahydrochloride salt. Yield 0.21 g (75%); ^1^H NMR (500 MHz, D_2_O): δ = 3.26 (t, *J* = 6.4, 4H), 3.58 (t, *J* = 6.4, 4H), 3.94 (s, 2H), 4.67 (s, 4H), 7.07–7.12 (m, 1H), 7.18–7.23 (m, 2H), 7.48–7.54 (m, 3H), 7.84 (s, 1H), 7.93–8.10 (m, 6H), 8.17–8.27 (m, 4H), 9.12 (d, *J* = 5.7, 2H); ^13^C RMN (125 MHz, D_2_O): δ = 45.9, 45.9, 49.5, 58.6, 120.0, 121.8, 124.2, 126.8, 126.9, 127.2, 127.4, 127.9, 129.2, 132.8, 131.5, 132.4, 132.8, 135.8, 136.6, 137.4, 144.1, 149.2, 149.3; HRMS (ESI+) *m*/*z*[M + H] calcd for C_35_H_35_N_5_: 526.2965, found: 526.2987. Anal. calcd for C_35_H_40_Cl_5_N_5_(H_2_O)_2_: C, 56.50; H, 5.96; N, 9.41, found: C, 56.57; H, 5.30; N, 9.37.

*1,7-Bis(6-methyl-1H-indazole)-4-(2-naphthylmethyl)-1,4,7-triazaheptane Pentahydrochloride* (**5**). 4-(2-naphthylmethyl)-1,4,7-triazaheptane (0.32 g, 1.31 mmol) was reacted with 1*H*-indazole-6-carboxaldehyde (0.38 g, 2.63 mmol) according to the general procedure described above. The residue was purified by column chromatography on neutral aluminum dioxide (CHCl_3_/MeOH 70:30) to give a brown oil, which then precipitated as its pentahydrochloride salt. Yield 0.15 g (6%); ^1^H NMR (500 MHz, DMSO-*d_6_*): δ = 7.99 (d, *J* = 0.6 Hz, 2H), 7.80 (dd, *J* = 16.5, 12.5 Hz, 4H), 7.61 (d, *J* = 8.3 Hz, 2H), 7.50–7.39 (m, 5H), 6.99 (dd, *J* = 8.3, 1.0 Hz, 2H), 3.72 (d, *J* = 12.8 Hz, 6H), 2.65–2.58 (m, 8H). DEPT ^13^C NMR (125 MHz, DMSO-*d_6_*): δ 133.3, 132.7, 127.9, 127.7, 127.3, 126.4, 125.9, 122.3, 121.4, 120.4, 59.4, 53.5, 46.9. MS (ESI+) *m*/*z*[M + H] 505.5.

### 2.2. In Vitro Assays

#### 2.2.1. *T. cruzi* Strains

Three different *T. cruzi* Arequipa strains—MHOM/Pe/2011/Arequipa, discrete typing unit (DTU) V [26]; IRHOD/CO/2008/SN3, DTU I [27]; TINF/CH/1956/Tulahuen, DTU VI [26]—were grown at 28 °C in Gibco^®^ RPMI 1640 Medium supplemented as previously reported [28], and used for in vitro screening.

#### 2.2.2. Screening against Extracellular Epimastigotes

Trypanocidal activity against epimastigotes was tested as previously reported [26]. In brief, 5 × 10^5^ mL^−1^ epimastigotes culture medium were treated by adding the compounds in serial dilutions from 100 to 0.2 µM in 96-well plates (200 µL·well^−1^) for 48 h. BZN and untreated growth control were also included in triplicate. Later, 20 μL of resazurin sodium salt (Sigma-Aldrich, St. Louis, MO, USA) was added, and the plates were incubated for another 24 h. Finally, the absorbance of the wells was measured at 570/600 nm and related to the control. The trypanocidal activity was expressed as the IC_50_ (inhibitory concentration 50) value using GraphPad Prism 6 software.

#### 2.2.3. Cytotoxicity Test

Cytotoxicity against mammalian Vero cells (EACC No. 84113001) was tested as previously reported [26]. In brief, 1.25 × 10^4^ Vero cells·mL^−1^, cultured at 37 °C, were treated by adding the compounds in serial dilutions from 400 to 1 µM in 96-well plates (200 µL·well^−1^) for 48 h. BZN and untreated controls were also included in triplicate. Later, 20 μL of resazurin sodium salt (Sigma-Aldrich) was added, and the plates were incubated for another 24 h. Finally, cell viability was determined following the same procedure as described to assess the trypanocidal activity in the epimastigotes.

#### 2.2.4. In Vitro Screening against Intracellular Amastigotes and Infected Cells

Trypanocidal activity against amastigotes was tested as previously reported [29]. In brief, bloodstream trypomastigotes (BTs), obtained by cardiac puncture from infected BALB/c mice [26], were used to infect 1 × 10^4^ Vero cells·well^−1^ in 24-well plates with rounded coverslips at a multiplicity of infection (MOI) ratio of 10:1. After 24 h of infection, non-internalized trypomastigotes were washed and the infected cells were treated by adding the compounds in serial dilutions from 50 to 0.5 µM in 500 mL·well^−1^ for 72 h. BZN and untreated growth controls were also included in triplicate. Later, the number of amastigotes and infected cells were determined in Giemsa stained preparations by analysing 500 host cells randomly distributed in microscopic fields. The trypanocidal activity was expressed as the IC_50_ using GraphPad Prism 6 software (version 6.07).

#### 2.2.5. In Vitro Screening against BTs

*T. cruzi* blood trypomastigotes were obtain by cardiac puncture from BALB/c albino mice during the parasitaemia peak after infection and diluted in RPMI (Gibco^®^) with 10% (*v*/*v*) FBS (heat-inactivated). Trypanocidal activity was performed in 96-well microtiter plates by seeding the parasites at 2 × 10^6^ mL^−1^, and after addition of the compounds at dosages of 50 to 0.5 µM, cultured in 200 µL/well volumes in a humidified 95% air, 5% CO_2_ atmosphere at 37 °C. BZN and untreated growth controls were also included in triplicate. After 24 h of incubation, 20 µL of Resazurin sodium salt (0.125 mg·mL^−1^) (Sigma-Aldrich) was added, and the plates were incubated for 4 h. Subsequently, the same process as described to determine the trypanocidal activity in epimastigote forms was followed.

### 2.3. In Vivo Assays on BALB/c Mice

#### 2.3.1. Ethics Statement

**Scheme 1:**
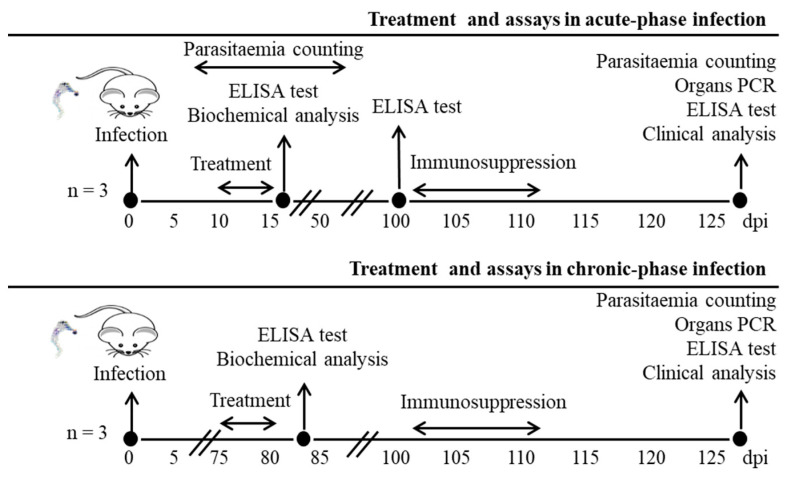
Timeline for all in vivo assays on BALB/c mice for the evaluation of compounds in the acute and chronic infection. dpi, day post-infection.

All animal work (Scheme 1) and maintenance was performed under RD53/2013 and approved by the Ethics Committee on Animal Experimentation (CEEA) of the University of Granada, Spain. Female BALB/c mice (aged 10–12 weeks and 20–22 g) were used in the subsequent in vivo assays.

#### 2.3.2. Infection and Treatment

Infection was carried out by intraperitoneal inoculation of 5 × 10^5^ BTs of *T. cruzi* Arequipa strain per mouse in 0.2 mL PBS [26]. The infection was monitored and on the 10th day post-infection (dpi), when BTs were visible in the bloodstream, the infection was confirmed.

Mice were divided into four groups (*n* = 3 per group): 0, negative control group (uninfected and untreated mice); I, positive control group (infected and untreated mice); II, BZN group (mice infected and treated with BZN); III, **15** group (mice infected and treated with **15**).

The treatment for mice treated in the acute and chronic CD began when the infection was confirmed (10th dpi) and was stablished that the animals had entered the chronic CD (75th dpi), respectively (Scheme 1) [29]. Compound **15** and BZN were orally administered for 5 consecutive days at a dose of 20 mg·Kg^−1^·day^−1^.

#### 2.3.3. Monitoring of Acute Parasitaemia by Counting

Parasitaemia levels were determined by counting BTs at 2–3 day intervals from peripheral blood drawn from the mandibular vein and diluted at a ratio of 1:100 [30]. The fresh blood microscopic examination was performed from the 7th day until the day when parasitaemia was not detected (Scheme 1).

#### 2.3.4. Infection Reactivation by Immunosuppression

After 100th dpi, once treatment was given to mice treated in the acute and chronic CD, immunosuppression was performed by the intraperitoneal injection of three doses of 200 mg·kg^−1^ of ISOPAC^®^ cyclophosphamide monohydrate (CP) at 3–4 day intervals [31] (Scheme 1). Within 7 days after the last injection, the reactivation rate was determined by counting BTs according the procedure described for parasitaemia levels in the acute CD and related to the control group [26].

#### 2.3.5. Blood Collection and Organs/Tissues Extraction

After CP-induced immunosuppression, the mice were euthanised using CO_2_ by exsanguination via cardiac puncture, and blood was collected. Then, 9 target organs/tissues were harvested [26]: adipose, bone marrow, brain, oesophagus, heart, lung, muscle, spleen, and stomach. These organs/tissues were perfused with pre-warmed PBS to avoid contamination with BTs [32], and stored at −80 °C. In addition, spleens were weighed to assess splenomegaly [26].

#### 2.3.6. Nested Amastigotes Detection by PCR

DNA extraction of the target organs/tissues was performed using Wizard^®^ Genomic DNA Purification Kit [29]. DNA was subjected to amplification by PCR based on the sequence of the *T. cruzi* splice leader (SL) intergenic region using two published primers (TC and TC1) that allow the amplification of a 300 base pairs fragment in different biological samples. The amplifications were performed using a MyCycler^TM^ Thermal Cycler (Bio-Rad, Hercules, CA, USA) using the commercial BioMix^TM^ (Bioline) with the following routine: 94 °C/4 min, 27 cycles of 94 °C/30 s, 55 °C/30 s, 72 °C/30 s, and 72 °C/5 min. Finally, the PCR products were resolved by electrophoresis on a 2% agarose gel containing GelRed nucleic gel stain.

#### 2.3.7. Immunoglobulin G Quantification by ELISA

Blood samples were collected in several dpi (Scheme 1) and processed according the method previously reported [26] to obtain serum. Serum samples were then aliquoted to ELISA test and clinical analysis.

ELISA test was performed in 96-well plates using diluted serum samples 1:80 in PBS, and excreted SOD [26] from epimastigotes as the antigen fraction. Finally, the absorbance of the wells was measured at 492 nm.

#### 2.3.8. Clinical Analysis

Heart, kidney and liver markers were measured from blood in several dpi (Scheme 1) with Cromakit^®^ as previously reported [26]. Mean and standard deviations were calculated using the levels measured for different populations of sera (*n* = 15, *n* = 3), and the confidence interval were also calculated based on a confidence level of 95% (100 × (1 − α) = 100 × (1 − 0.05)).

### 2.4. MoA Tests

#### 2.4.1. Excreted Metabolites by ^1^H Nuclear Magnetic Resonance (NMR)

5 × 10^5^ mL^−1^ epimastigotes culture medium were treated by adding the compounds at IC_25_ concentrations in 25 cm^2^ cell culture flasks for 72 h. Untreated controls were also included in triplicate. Cultures were then centrifuged and filtered, and the metabolites of the supernatants were measured by ^1^H NMR (VARIAN DIRECT DRIVE 500 MHz Bruker) with AutoX probe, D_2_O as solvent and 2,2-dimethyl-2-silapentane-5-sulphonate as the reference signal [33]. Analyses were conducted as previously reported [34].

#### 2.4.2. Mitochondrial Membrane Potential by Flow Cytometry

Epimastigotes of *T. cruzi* Arequipa strain described in the NMR analysis were collected by centrifugation, washed three times in PBS and stained with 10 mg·mL^−1^ rhodamine 123 (Rho) dye (Sigma-Aldrich) in 0.5 mL PBS for 20 min [35]. Control epimastigotes with a fully depolarized mitochondrion were obtained by incubation for 40 min with 10 mM KCN prior to Rho loading [36]. Non-stained parasites were also included in triplicate. After incubation, epimastigotes were immediately processed and analysed by flow cytometry as previously reported [34].

#### 2.4.3. SOD Inhibition Tests

The activities of either excreted Fe-SOD—obtained as previously reported [37]—and commercial Cu/Zn-SOD from human erythrocytes (Sigma-Aldrich) exposed to the compounds at a concentration range from 100 to 0.1 µM were determined using the Beyer and Fridovich method [38].

#### 2.4.4. Docking Methods

The compound **15** was designed with the program Avogadro, in the protonation state at pH 7.4 calculated by this program (pubmed/22889332). The Chimera software was used for calculating the compound AM1 charges (pubmed/15264254). The structure of the mytochondrial *T. cruzi* Fe-SOD protein, with Protein Data Bank (PDB) code 4DVH, was selected as a target for the docking (PubMed: 24616096). The residue numbering used here corresponds to that of the PDB, with the uniprot Q4DCQ3 entry numbering between parentheses. The program PDB2PQR was used to calculate the protein protonation state at pH 7.4 (pubmed/17488841), and the Autodock4.0 program was used for calculating Gasteiger charges and performing the docking (pubmed/17274016). The docking was performed with the Lamarckian genetic algorithm (LGA) and using a grid centered on the dimer interface (pubmed/28002965). 100 docking runs were performed and the one with best score energy was selected as potential binding of the compound to the SOD enzyme.

### 2.5. Statistical Analyses

The statistics were performed using SPSS software (v. 21, IBM). T-test for paired samples (*p* < 0.05 and 95% confidence level) ans contingency tables (prevalence) were conducted.

## 3. Results

### 3.1. Chemistry

**Scheme 2:**
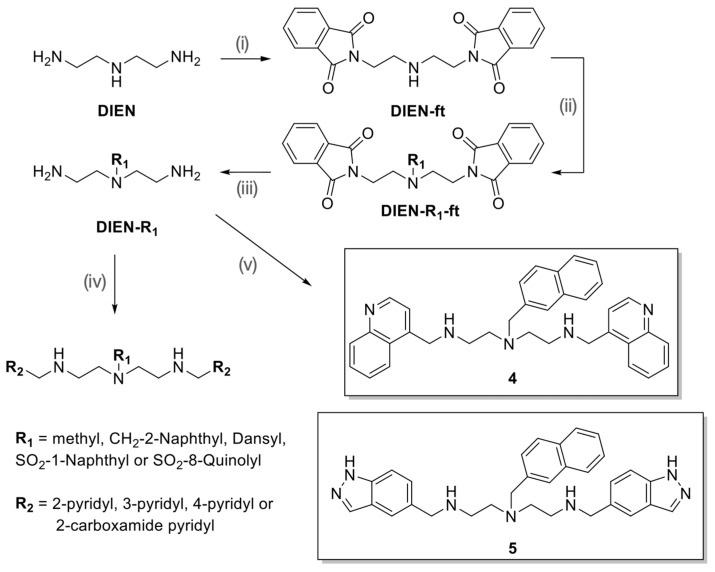
Synthesis of **1**–**13**. Reagents and conditions: (**i**) phthalic anhydride (2.2 equiv), CH_3_COOH, reflux, 1.5 h; (**ii**) 2-naphthylCH_2_Br or R_1_SO_2_Cl (R_1_ = dansyl, naphthyl, 8-quinolyl), K_2_CO_3_ (1.6 equiv), CH_3_CN, reflux, 24 h; (**iii**) N_2_H_4_, EtOH, reflux, 24 h. (**iv**) pyridyl carboxaldehyde, EtOH, 4 h; NaBH_4_ (10 equiv), EtOH, 24 h. (**v**) 4-quinoline carboxaldehyde or with 1*H*-indazole-6-carboxaldehyde, EtOH, 4 h; NaBH_4_ (10 equiv), EtOH, 24 h.

**Scheme 3:**
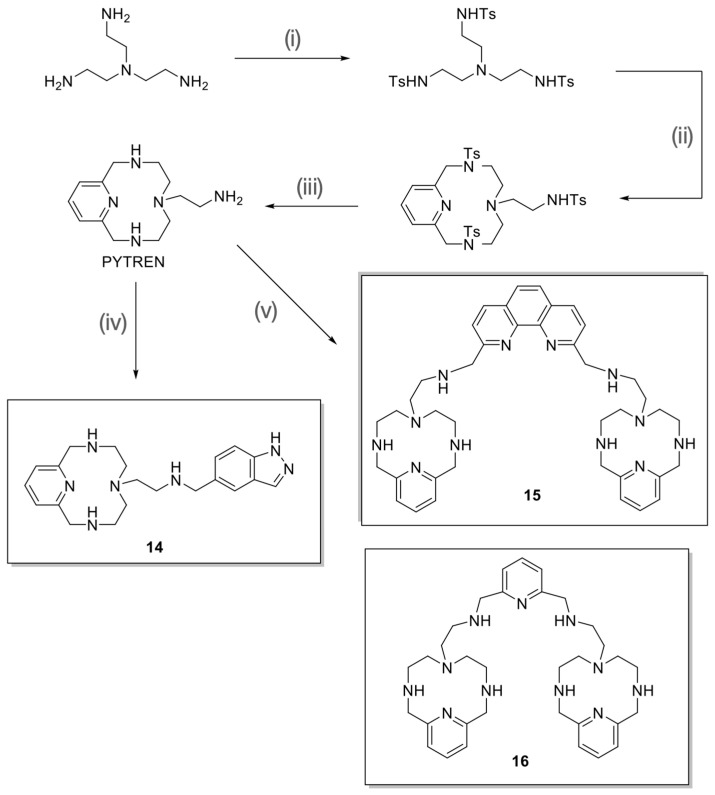
Synthesis of **14**–**16**. Reagents and conditions: (**i**) tosyl chloride (3 equiv), K_2_CO_3_ (3 equiv), THF, 24 h; (**ii**) 2,6-bisbromomethylpyridine, K_2_CO_3_ (3 equiv), CH_3_CN, reflux, 24 h; (**iii**) phenol (3 equiv), hydrogen bromide solution in acetic acid (3 equiv), reflux, 24 h; (**iv**) 1H-indazole-6-carboxaldehyde, EtOH, 4 h; NaBH_4_ (10 equiv), EtOH, 24 h; (**v**) pyridine-2,6-dicarboxaldehyde or 1,10-phenanthroline-2,9-dicarboxaldehyde, EtOH, 4 h; NaBH_4_ (10 equiv), EtOH, 24 h.

**Scheme 4:**
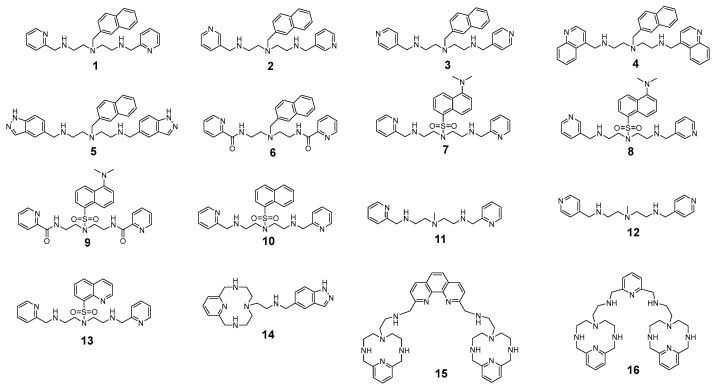
Chemical structures of compounds **1**–**16**.

An inexpensive synthetic strategy has been developed to prepare a library of polyamine compounds based on the commercially available 1,4,7-triazaheptane (DIEN) (Scheme 2). Previously, we explored the functionalization at the 4 position of DIEN with methyl, 2-methyl-naththyl, dansyl, 1-naphthylsulfonyl and 8-quinolylsulfonyl groups combined with the pyridine substitution at 1 and 7 positions of 1,4,7-triazaheptane. Briefly, the general synthesis embraces four synthetic steps. Initially, the terminal amino groups (1 and 7 positions) of DIEN were protected with an excess of phthalic anhydride (2.2 equiv) to afford the protected amine DIEN-ft. The central nitrogen was alkylated or sulfonylated by nucleophilic attack of the corresponding alkyl or arylsulfonyl halides to afford the 3-N-substituted diphthalimides. Then, the protecting groups were removed with hydrazine in ethanol to give the free alkylated polyamines. In the last step, the alkylated polyamines were reacted with pyridinecarboxaldehydes and the corresponding imines were subsequently reduced in situ with NaBH_4_ to afford the final compounds. The fully characterization of the compounds **1**–**3** and **6**–**13** can be found in the corresponding published works [21,22,23,24]. In order to expand our library of DIEN based molecules containing larger aromatic groups than pyridyl at the 1 and 7 positions of DIEN, we reacted the 4-(2-naphthylmethyl)-1,4,7-triazaheptane with the corresponding carboxaldehydes of quinoline and indazole moieties to afford the compounds **4** and **5** (Scheme 2). These new compounds (Scheme 3) were fully characterised by NMR (^1^H, ^13^C and DEPT), high-resolution mass spectrometry (HRMS) and elemental analysis. Compounds **14**–**16** were prepared through the condensation of the corresponding aldehyde of indazole, phenanthroline or pyridine with the polyazamacrocycle PYTREN (Scheme 3) followed by the reduction with NaBH_4_ to afford the compounds **14**–**16**. The compounds characterization can be found in references [24,25]. Scheme 4 shows the chemical structures of compounds **1**–**16**.

### 3.2. In Vitro Evaluation

The results, expressed as IC_50_ and SI, are summarized in Table 1 and Table 2, respectively. The reference drug BZN was included to compare activities. Compounds **2**, **8**, and **15** were chosen as hit compounds after the first screening against epimastigotes. Interestingly, compound **15** showed higher IC_50_ and SI values than BZN against all *T. cruzi* forms in all three parasite variants evaluated, devoid of drug resistance. Hence, compound **15** was prioritised as lead compound for in vivo evaluation after the second screening against the forms developed in vertebrate hosts and responsible for the acute and chronic infections [39].

**Figure 1:**
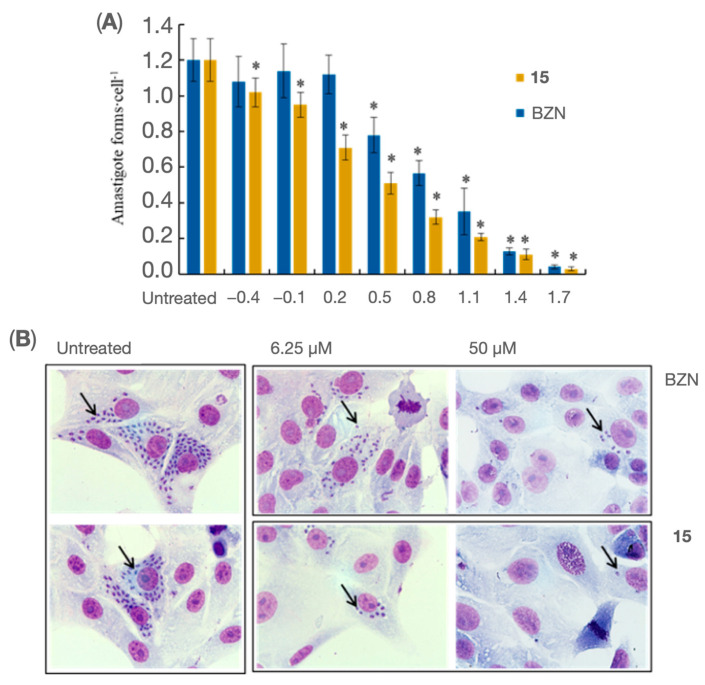
(**A**) Number of *Trypanosoma cruzi* amastigotes per Vero cell exposed to compound **15** and the reference drug benznidazole (BZN). Values are the mean of three separate determinations ± standard deviation. * Significant differences between untreated and treated amastigotes (*p* < 0.05). (**B**) Representative Giemsa stained images of untreated and treated Vero cells infected. Arrows point to intracellular amastigote forms of *T. cruzi* Arequipa strain.

Before going ahead on in vivo evaluation, the average number of amastigotes per cell was also determined after 72 h of exposure at different concentrations of compound **15** and BZN (Figure 1), giving an idea of the killing rate. It is well-known that the eradication of parasites as soon as possible after infection can prevent serious disease, and sterile cure is a new essential topic in CD to prevent parasite reproliferation [40]. This topic has been notably supported, especially after the failure of the lastest candidates—posaconazole and ravuconazole—in clinical trials [3,41]. Currently, BZN is considered as a fast-acting and cidal drug, showing a reduction in the number of amastigotes to practically zero at 50 μM (Figure 1). Interestingly, compound **15** exhibited behaviour similar to BZN at 50 μM, but its cidal effect was observed from lower concentrations.

### 3.3. In Vivo Evaluation

Compound **15** was selected as lead compound for in vivo evaluation in BALB/c mice. Considering the topics stablished for the TPP [3,40,42], trials were conducted to evaluate the efficacy of compound **15** after oral administration in both the acute and chronic infection according to the experiments stablished in the Scheme 1. Curing chronic infections are the primary clinical need [43]; however, most in vivo evaluations have focused on acute infections because it is easier to monitor parasite burden [44,45]. Here, we highlight that chronic infections should be the main research focus.

Compound **15** and BZN were orally administered for 5 consecutive days at a dose of 20 mg·Kg^−1^·day^−1^. It is defined that a compound exhibiting a reasonable parasitaemia reduction after a treatment schedule of 5 consecutive days can be taken as a candidate compound [8]. In addition, the treatment represents a subcurative dose of BZN to demonstrate whether or not compound **15** was more effective than reference drug.

**Figure 2:**
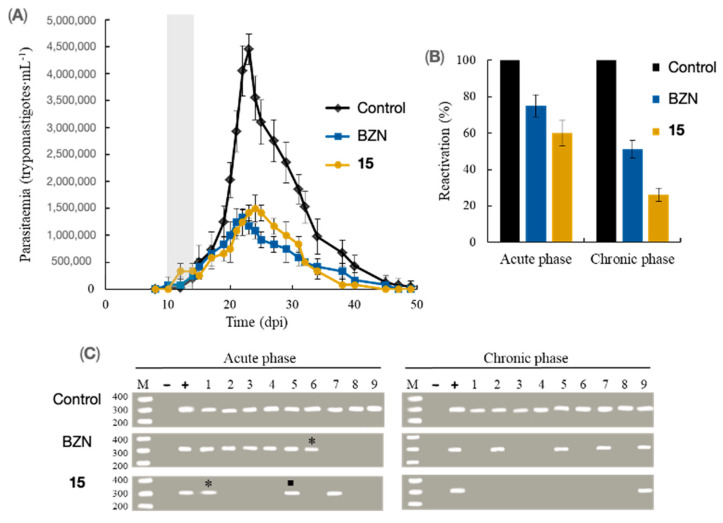
(**A**) Parasitaemia profile of untreated and treated mice during the acute infection. Treatment time is represented in grey. Values are the mean of three mice ± standard deviation. Significant differences between untreated and treated mice (*p* < 0.05). (**B**) Reactivation of parasitaemia after immunosuppression of mice during the chronic infection. Values are the mean of three mice ± standard deviation. Significant differences between untreated and treated mice (*p* < 0.05). (**C**) PCR analysis of the nine target organs/tissues after treatment of mice during the acute and chronic infection. Lanes: M, base pair marker; −, PCR negative control; +, PCR positive control; 1, adipose; 2, bone marrow; 3, brain; 4, oesophagus; 5, heart; 6, lung; 7, muscle; 8, spleen; 9, stomach. * 1/3 of the mice showed PCR product on electrophoresis; ^■^ 2/3 of the mice showed PCR product on electrophoresis. BZN, benznidazole.

First, the parasitaemia profile of the mice treated during the acute infection were measured (Figure 2A). Control mice showed detectable parasitaemia up to 50 dpi, and suffered the parasitaemia peak approximately on 25 dpi. BZN- and compound **15**-Treated mice experienced a considerable parasitaemia reduction: they exhibited a lower parasitaemia burden throughout the post-treatment period, showed a ~65% reduction on the day of maximum parasitaemia, and their parasitaemia were undetectable 3 and 5 days before, respectively. It is noteworthy that compound **15**-treated mice showed a considerable trend change in the parasitaemia curve during the treatment period, suggesting that longer treatment periods could clear the parasitaemia in the acute infection.

Second, the experimental cure of mice treated in both the acute and chronic infection was determined using a double checking based on (a) parasitaemia reactivation after immunosuppression (Figure 2B) and (b) PCR of the target organs/tissues (Figure 2C). This double checking is widely used in animal models to evaluate the treatment effectiveness: animals whose parasitaemia reactivation does not reappear and show negative PCR in target organs/tissues are considered cured [26,46,47,48,49]. Immunosuppression is a useful method to demonstrate cure since apparently cured immunocompromised mice show parasitaemia reactivation, and any remaining parasite is responsible for this reactivation [46]. Similarly, seemingly cured immunocompromised patients subjected to transplantation, treated with anticancer drugs or diagnosed with AIDS, suffer from a clinically aggressive parasitaemia reactivation [47].

As observed, BZN- and compound **15**-treated mice reduced the parasite burden after treatment in both the acute and chronic infection. It is highlighted that compound **15** showed higher efficacy than the reference drug BZN: compound **15** achieved (a) a reactivation of 60% and 26% after treatments in the acute and chronic infections (75% and 51% of reactivation for BZN, respectively) (Figure 2B), and (b) a clearance of nested parasites of 67% and 89% in target organs/tissues after treatments in the acute and chronic infections (33% and 56% of parasite-free organs/tissues for BZN, respectively) (Figure 2C). Hence, we can state that compound **15** show the criteria stablished for potential compounds, that is, parasite burden reduction >BZN (>80% for chronic treatment) and efficacy in both acute and chronic infection after oral administration.

Third, the immune response to *T. cruzi* infection of mice treated in both the acute and chronic infection was assessed by quantifying immunoglobulin G (Ig G) (Figure S7) and splenomegaly (Figure S8). They are manifested in both the acute and chronic infections, and allow an indirect evaluation of the treatment efficacy since they are directly associated with the parasitic load [26,29,38]. In short, the results suggest that BZN- and compound **15**-treated mice reduced the parasite load with respect to untreated control mice, being the treatment with compound **15** the most effective again.

Finally, toxic effects associated with the treatment in both the acute and chronic infections were analysed by measuring heart, kidney and liver markers (Table S1), including values for uninfected mice. Most markers showed alterations in the samples obtained 2 days after the administration of both compounds (BZN and compound **15**), but they returned to normal levels on the necropsy day. In addition, none of the mice died or lost more than 10% body mass. The low toxicity exhibited by compound **15** allows it to be tested at higher doses (partial or total) in order to reach a sterile cure, as stated above.

### 3.4. MoA Evaluation

**Figure 3:**
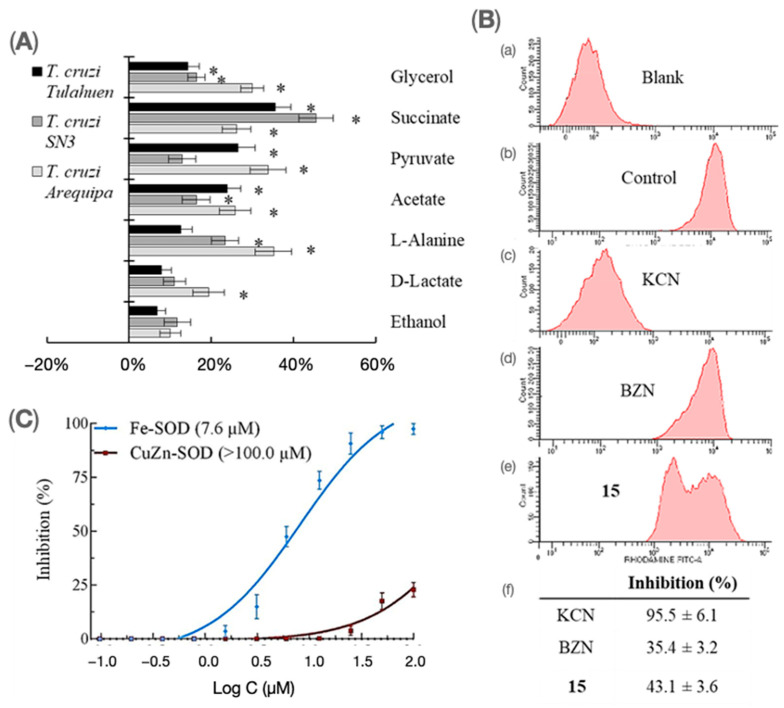
(**A**) Catabolites excreted by *Trypanosoma cruzi* epimastigotes exposed to **15** at IC_25_ concentrations in comparison to untreated epimastigotes. Values are the mean of three separate determinations ± standard deviation. * Significant differences between untreated and treated epimastigotes (*p* < 0.05). (**B**) Mitochondrial membrane potential from epimastigotes of *Trypanosoma cruzi* Arequipa strain exposed at IC_25_ concentrations: (**a**) blank, (**b**) control (untreated), (**c**) potassium cyanide (KCN), (**d**) BZN, (**e**) **15**, (**f**) Inhibition on mitochondrial membrane potential with respect to untreated epimastigotes. Values are the mean of three separate determinations ± standard deviation. Significant differences between untreated and treated epimastigotes (*p* < 0.05). (**C**) Inhibition of *Trypanosoma cruzi* Fe-SOD—activity 42.0 ± 3.8 U·mg^−1^—and human erythrocytes CuZn-SOD—activity 47.3 ± 4.1 U·mg^−1^—for **15**. Values are the mean of three separate determinations ± standard deviation. In brackets: IC_50_ value.

Here, we evaluate the MoA of compound **15** from an energetic viewpoint, testing the two main organelles involved for this purpose in *T. cruzi*—glycosome and mitochondrion—since its activity could be explained by a bioenergetic collapse. So that, treated *T. cruzi* epimastigotes were analysed by ^1^H RMN and flow cytometry in order to measure different glucose metabolism catabolites (Figure 3A) and the mitochondrial integrity (Figure 3B), respectively.

As shown, the excretion of all catabolites from compound **15**-treated parasites was notably increased concerning the corresponding untreated parasites. Succinate and pyruvate were the most increased excretions, which are likely to be result of a decreased ATP synthesis due to mitochondrial dysfunction caused by redox stress [50]. Moreover, owing to the major role of succinate is to sustain the redox balance via NADH reoxidation produced in the glycosome catabolic route, it is feasible that this pathway can be enhanced to sustain the balance, with the subsequent increase in succinate as the final product [50,51].

Consequently, the mitochondrial membrane potential (Figure 3B) and the enzyme Fe-SOD inhibition—one of the most relevant therapeutic targets [52]—were evaluated (Figure 3C). As observed, BZN-treated parasites reduce the mitochondrial membrane potential (35.4%) due to BZN MoA [53,54]. Depolarisation was even higher in compound **15**-treated parasites (43.1%). Alternatively, compound **15** was found to selectively inhibit the *T. cruzi* Fe-SOD enzyme. All this lead us to hypothesize that the cidal activity of compound **15** can be attributed to a mitochondria-dependent bioenergetic collapse and redox stress by Fe-SOD inhibition.

**Figure 4:**
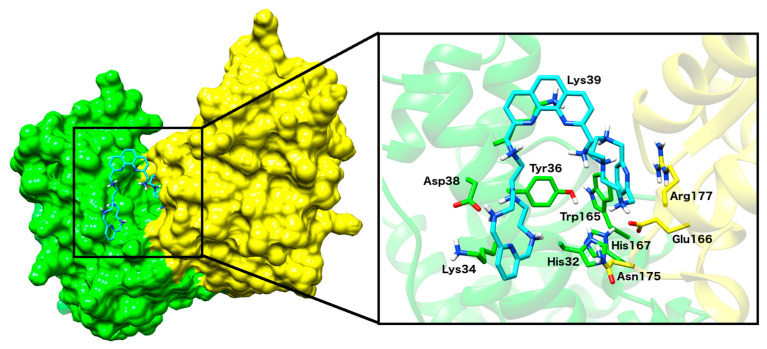
Proposed binding mode of compound **15** to the mitochondrial Fe-SOD enzyme (PDB ID 4DVH) (PubMed: 24616096). In the (**left**), the protein surface is shown, with one protomer in green and the other in yellow, and the compound as cyan sticks bound in the dimer interface. In the (**right**), we can see a close-up of the binding site. The following color scheme was used in the whole panel: oxygen (red), nitrogen (blue), hydrogen (white), and carbon (protein chain A in yellow and chain B in green-, compound **15** in cyan, orange or pink). The figures were created by the software Chimera.

The docking study, performed with Autodock4.0, suggests that the compound binds in the dimer interface of the SOD enzyme. In the best scored pose (Figure 4) we observe one of the side macrocycles of the compound binding deeo in the dimer interface, presenting electrostatic interactions with Arg177 (Arg208 in uniprot), His32 (His63 in uniprot) and Asn175 (Asn206 in uniprot). The central aromatic moiety of the compound is suggested to present electrostatic interactions with Lys39 (Lys70 in PDB), and the second side macrocycle with Asp38 (ASP69 in PDB).

## 4. Discussion

New drug development should define a clear TPP. In this framework, Drugs for Neglected Diseases *initiative* (DND*i*) convened a multidisciplinary group of experts in 2010 to provide a TPP for CD [7]. Recently, another TPP has been reported [40], although both are quite similar. As far as the early drug discovery is concerned, several topics must be taken into account: parasite genetic diversity (including intra-DTU), drug resistance, and sensitivity against moieties [7,40]. Concerning the in vivo activity profile, drugs must be highly effective in both the acute and chronic infections (even achieving the so-called sterile parasitological cure), allow oral administration and a simple treatment regimen, and be safe and well tolerated [3,7,40]. Recently, the drug candidate posaconazole has failed in clinical trials because of the difficulties in achieving sterile cure [55].

Alternatively, the adoption of balanced selection criteria is an important aspect in DDP, although in the last 10 years no single criterion has been adopted by different experts [8,15,16,17,18,19]. In short, we can state that potential candidates must show for (a) in vitro models: higher trypanocidal than BZN in amastigotes and trypomastigotes (preferably IC_50_ < 10 µM), maximum activity >90%, activity against a wide panel of variants (including BZN-resistant strains), and selectivity >10 (preferably >100); and (b) in vivo models: parasite burden reduction ≥BZN (preferably >80% or parasitological cure), efficacy in both the acute in chronic CD, oral efficacy, and no toxicity.

The development of screening cascade—using a phenotypic-based approach—and the establishment of progression criteria for compounds were as follows: (a) three different parasite variants—including a BZN-resistant strain—belonging to different DTUs, with different locations, hosts and tropisms, were used to evaluate the compounds with good performance; (b) mammalian Vero cells were used to determine the compounds effect on the viability; (c) epimastigotes were used for a first screening because of their simple culture, selecting compounds with IC_50_ values < 20 µM and selectivity indexes (SI) > 10; (d) amastigotes and trypomastigotes—developed forms in vertebrate hosts—were then used for a second screening, selecting compounds with IC_50_ values < 10 µM (or close), SI > 50, and maximum activity > 90% for all strains tested. Regards to the clinic, it has been postulated that inappropriate pharmacokinetics/pharmacodynamics (PK/PD) between current drugs and tissue location of parasites is linked to the inability to reliably cure chronic infections [56,57]. Currently, new treatment schedules are a major challenge [44].

Here, we present compound **15** as a candidate compound for an alternative therapy for CD as it meets the selection criteria stablished for in vitro and in vivo models. As observed, compound **15** showed promising in vivo results after an early preclinical trial: oral administration for 5 days at subcurative doses. New treatment schedules—higher doses and/or longer treatment guidelines—should be studied in order to achieve a sterile cure. A synergistic treatment represents another alternative that should be explored, preferably using a combination of compounds with different MoA [58,59].

When considering compounds for CD, Chatelain highlighted that candidate compounds must show a fast and cidal MoA [8]. The latest candidate compounds—posaconazole and ravuconazole—exhibited excellent activity in both in vitro and in vivo models, but they failed in humans [41]. Further studies confirmed that these drugs maintain residual quiescent amastigotes after treatment, but viable and infective [60,61]. The MoA of these drugs is responsible for clinical failure: they are ergosterol biosynthesis inhibitors, which mean that they inhibit the proliferation of the *T. cruzi* replicative stages; hence, the non-replicative quiescent amastigotes are insensitive to these drugs [62,63]. From here, the MoA is an important factor. Here, we present compound **15** whose cidal activity can be attributed to a mitochondria-dependent bioenergetic collapse and redox stress by Fe-SOD inhibition. The possibility of multitarget activity should however not be rejected.

## 5. Conclusions

16 aryl polyamines were screened against *T. cruzi*. After a complete phenotypic-based screening in both in vitro and in vivo models, we identified compound **15** as a promising alternative that represent a step forward to combat acute and chronic CD. Its high activity profile could be ascribed to a mitochondria-dependent bioenergetic collapse and redox stress by inhibition of the Fe-SOD enzyme, causing *T. cruzi* death. Given that the ultimate goal is to achieve a sterile parasitological cure, long-term treatments, new treatment schedules based on pharmacokinetic/pharmacodynamic studies or even combined therapies compound **15**-BZN should be exploited.

## Figures and Tables

**Table 1:** Activity of benznidazole and compounds tested against the three developmental forms of *Trypanosoma cruzi*, and toxicity on mammalian Vero cells.

Compound	Activity IC_50_ (µM) ^a^			Activity IC_50_ (µM) ^a^			Activity IC_50_ (µM) ^a^			Toxicity IC_50_ (µM) ^b^ Vero Cell
	*T. cruzi* Arequipa Strain			*T. cruzi* SN3 Strain			*T. cruzi* Tulahuen Strain			
	E	A	T	E	A	T	E	A	T	
**BZN**	16.9 ± 1.8	8.3 ± 0.7	12.4 ± 1.1	36.2 ± 2.4	16.6 ± 1.4	36.1 ± 3.1	19.7 ± 1.7	10.0 ± 0.8	15.1 ± 1.3	80.4 ± 7.1
**1**	28.3 ± 2.4	nd	nd	27.1 ± 2.1	nd	nd	26.0 ± 2.7	nd	nd	58.1 ± 6.0
**2**	2.9 ± 0.3	6.2 ± 0.6	4.8 ± 0.5	5.7 ± 0.5	nd	nd	15.8 ± 1.4	nd	nd	38.5 ± 4.1
**3**	18.7 ± 1.6	nd	nd	21.8 ± 1.9	nd	nd	19.2 ± 1.7	nd	nd	25.8 ± 2.7
**4**	25.2 ± 2.7	nd	nd	11.0 ± 1.0	nd	nd	22.6 ± 3.3	nd	nd	5.3 ± 0.4
**5**	31.3 ± 3.1	nd	nd	33.8 ± 3.1	nd	nd	25.3 ± 2.1	nd	nd	12.4 ± 1.4
**6**	12.0 ± 1.1	nd	nd	10.0 ± 1.2	nd	nd	16.1 ± 1.7	nd	nd	63.2 ± 5.7
**7**	11.5 ± 1.1	nd	nd	10.4 ± 1.1	nd	nd	12.1 ± 1.1	nd	nd	41.3 ± 3.7
**8**	48.1 ± 3.9	nd	nd	36.4 ± 3.4	nd	nd	10.0 ± 1.1	17.0 ± 1.5	12.2 ± 1.2	136.3 ± 14.8
**9**	12.4 ± 1.2	nd	nd	59.6 ± 6.2	nd	nd	39.3 ± 4.2	nd	nd	49.3 ± 5.0
**10**	15.2 ± 1.8	nd	nd	14.3 ± 1.8	nd	nd	12.9 ± 1.8	nd	nd	14.9 ± 1.3
**11**	12.8 ± 1.7	nd	nd	21.4 ± 2.0	nd	nd	30.0 ± 2.7	nd	nd	23.3 ± 2.1
**12**	104.0 ± 12.8	nd	nd	87.5 ± 9.0	nd	nd	95.2 ± 8.4	nd	nd	187.7 ± 20.4
**13**	12.7 ± 1.4	nd	nd	5.5 ± 4.9	nd	nd	21.0 ± 1.8	nd	nd	28.3 ± 2.4
**14**	18.3 ± 1.4	nd	nd	25.8 ± 2.7	nd	nd	22.4 ± 2.1	nd	nd	60.3 ± 7.0
**15**	6.4 ± 0.6	2.5 ± 0.3	1.6 ± 0.1	16.9 ± 1.4	11.3 ± 0.9	10.6 ± 0.9	11.4 ± 1.1	6.8 ± 0.7	7.8 ± 0.7	654.9 ± 51.9
**16**	102.4 ± 9.8	nd	nd	155.0 ± 14.9	nd	nd	129.3 ± 14.7	nd	nd	135.8 ± 14.7

^a^ IC_50_, inhibition concentration 50: concentration (µM) required to inhibit 50% population, determined using GraphPad Prism 6. ^b^ Towards Vero cells. The value is the mean of three separate determinations ± standard deviation. E, epimastigotes; A, amastigotes; T, trypomastigotes; BZN, benznidazole; nd, not determined.

**Table 2:** Selectivity index for benznidazole and selected compounds against the three developmental forms of *Trypanosoma cruzi*.

Comp.	Selectivity Index ^a^ *T. cruzi* Arequipa Strain			Selectivity Index ^a^ *T. cruzi* SN3 Strain			Selectivity Index ^a^ *T. cruzi* Tulahuen Strain		
	E	A	T	E	A	T	E	A	T
**BZN**	4.8	9.7	6.5	2.2	4.8	2.2	4.1	8.1	5.3
**2**	13.5 (3)	6.2 (1)	8.0 (1)	6.7 (3)	nd	nd	2.4 (1)	nd	nd
**8**	2.8 (1)	nd	nd	3.7 (2)	nd	nd	13.7 (3)	8.0 (1)	11.2 (2)
**15**	101.7 (21)	262.0 (27)	409.3 (63)	38.7 (18)	58.0 (12)	62.0 (28)	57.4 (14)	96.3 (12)	83.6 (16)

^a^ Selectivity index (SI), IC_50_ Vero cells/IC_50_ developmental form of the parasite. In brackets: number of times that compound exceeds the reference drug SI. E, epimastigotes; A, amastigotes; T, trypomastigotes; nd, not determined.

## Data Availability

Not applicable.

## Supplementary Materials

The following supporting information can be downloaded at: https://www.mdpi.com/article/10.3390/pharmaceutics15010140/s1, Figure S1. ^1^H NMR spectra of compound **4**; Figure S2. Homonuuclear correlation ^1^H-^1^H COSY spectra of compound **4**; Figure S3. ^13^C NMR spectra of compound **4**; Figure S4. Heteronuclear correlation ^1^H-^13^C HSQC spectra of compound **4**; Figure S5. ^1^H NMR spectra of compound **5**; Figure S6. DEPT NMR spectra of compound **5**; Figure S7. Anti-*Trypanosoma cruzi* immunoglobulin G levels; Figure S8. Weight percentage of spleens; Table S1. Biochemical clinical analysis.
