# Supplementary material for: Identification of Aryl Polyamines Derivatives as Anti-Trypanosoma cruzi Agents Targeting Iron Superoxide Dismutase
Source: Pharmaceutics. 2022 Dec 31;15(1):140. doi: 10.3390/pharmaceutics15010140 (PMC9863987; doi:10.3390/pharmaceutics15010140)
Supplement: Supplementary file 1 [file pharmaceutics-15-00140-s001.zip › pharmaceutics-2043593-supplementary.pdf]

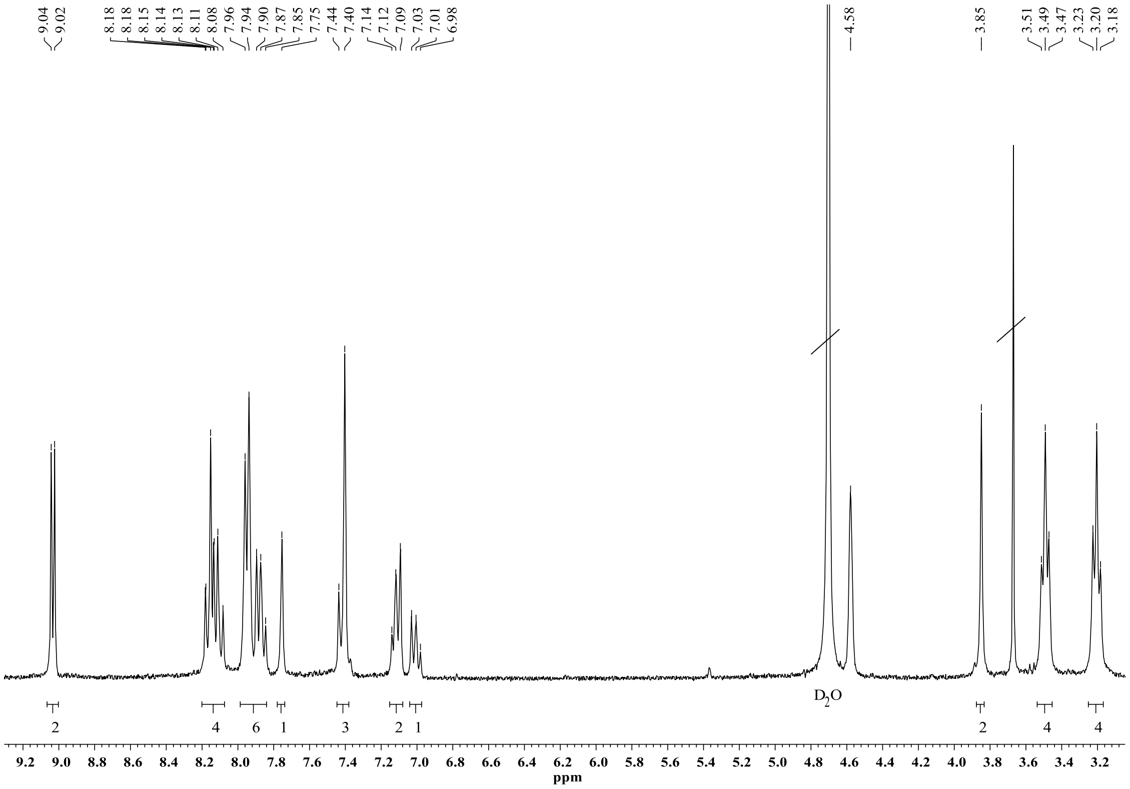

Figure S1. ^{1}H NMR spectra of compound **4**.

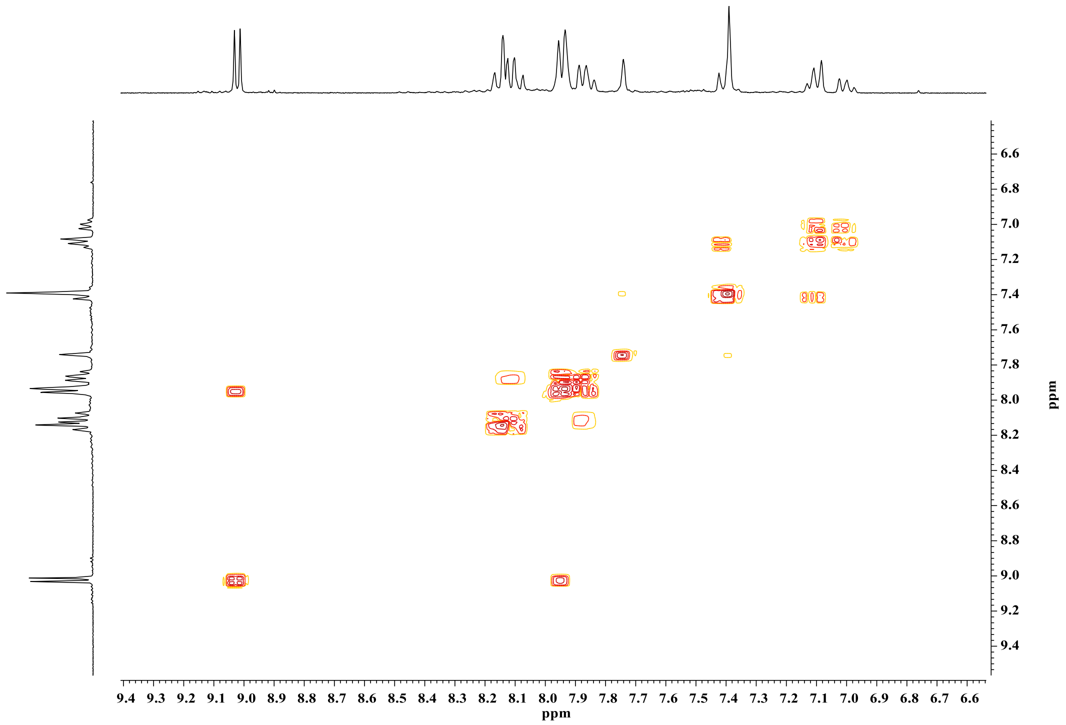

Figure S2. Homonuclear correlation ^{1}H-^{1}H COSY spectra of compound **4**.

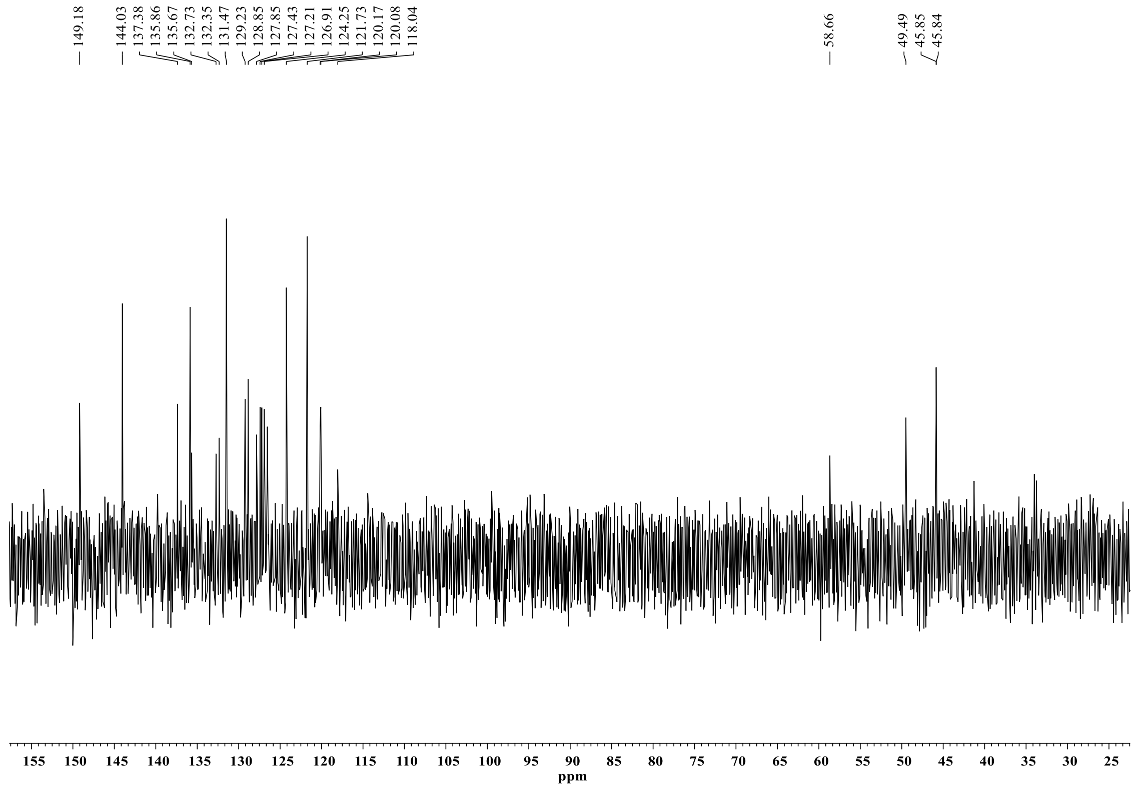

Figure S3. ^{13}C NMR spectra of compound **4**.

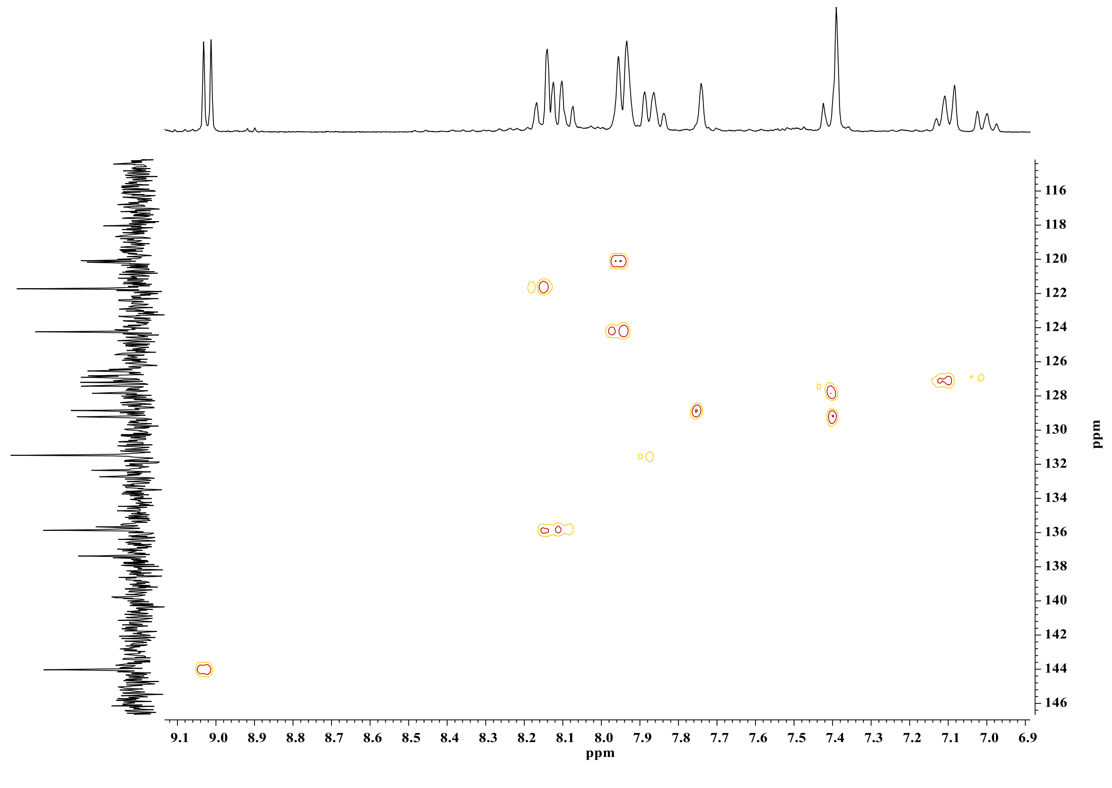

Figure S4. Heteronuclear correlation ^{1}H-^{13}C HSQC spectra of compound **4**.

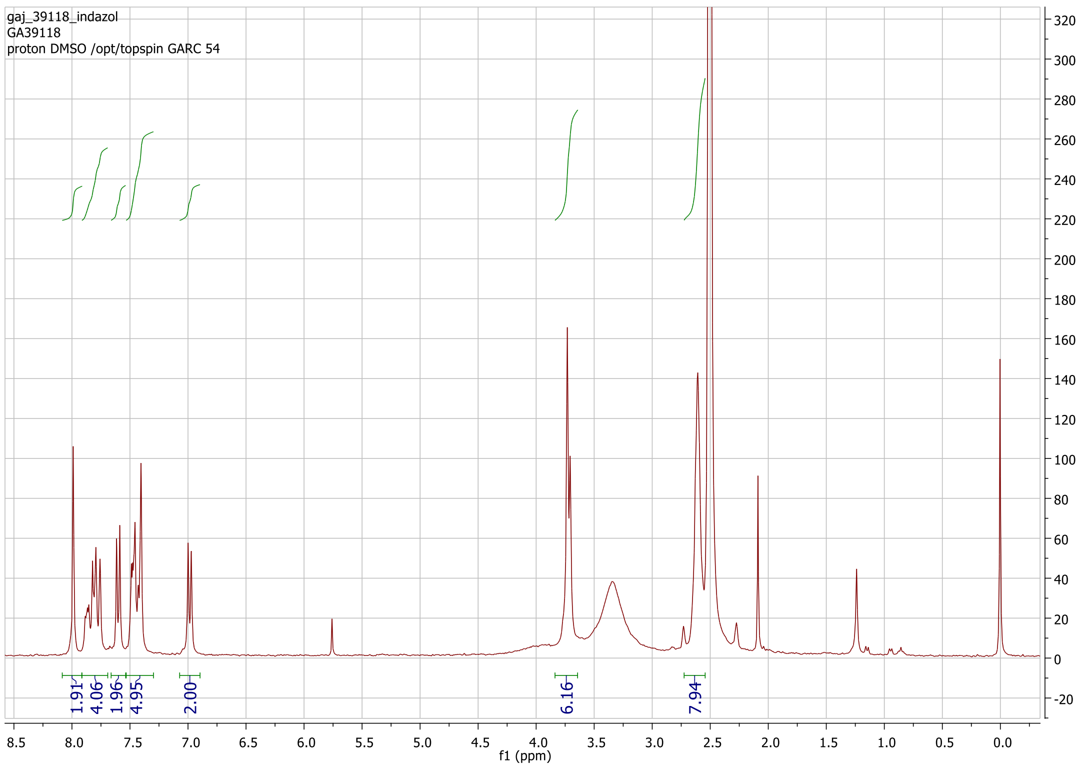

Figure S5. ¹H NMR spectra of compound **5**.

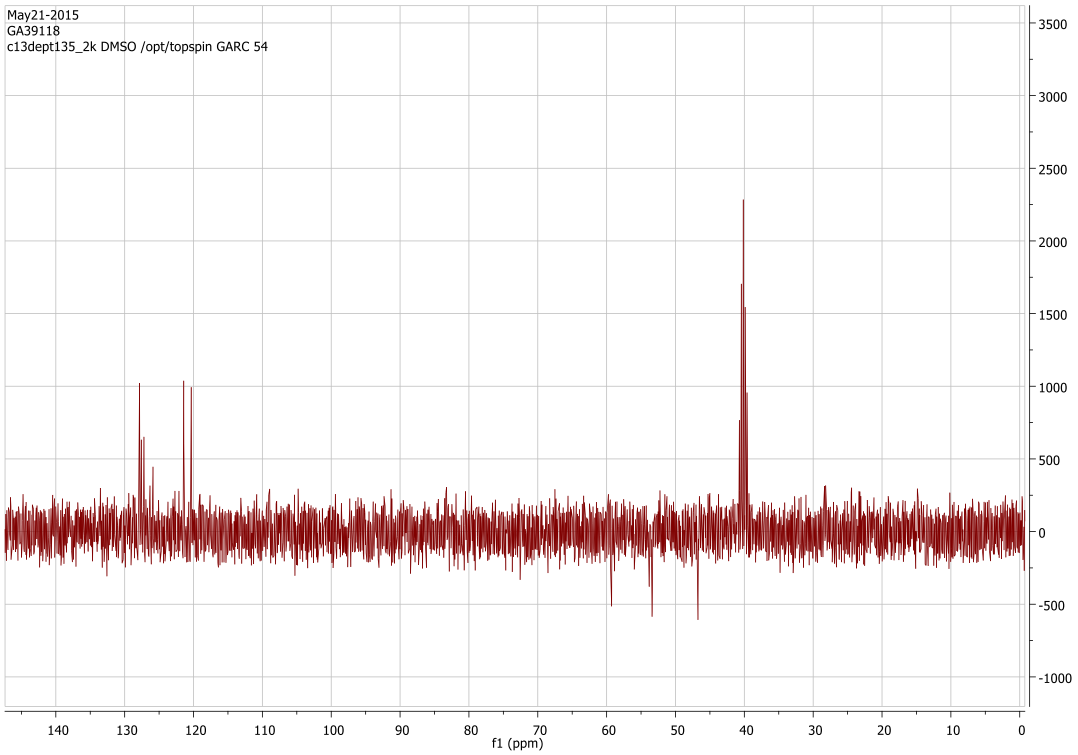

Figure S6. DEPT NMR spectra of compound **5**.

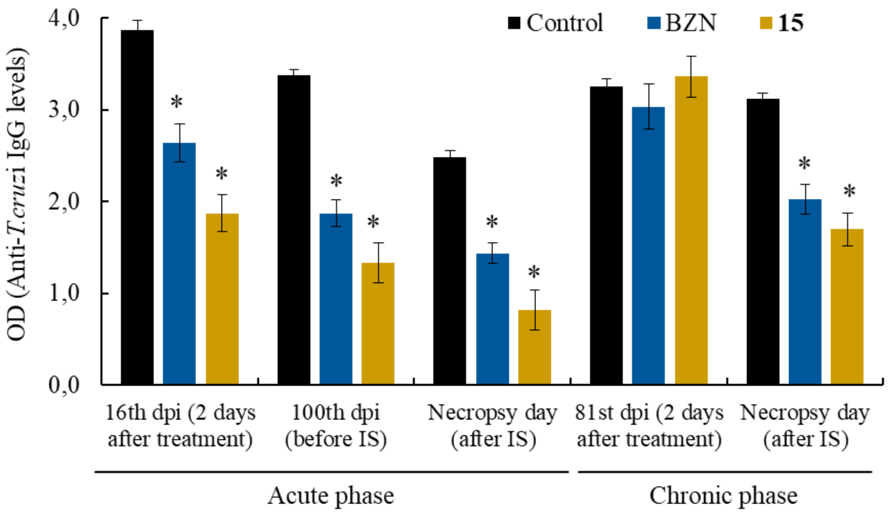

Figure S7. Anti-*Trypanosoma cruzi* immunoglobulin G (IgG) of mice treated during the acute and chronic infection: control, benznidazole (BZN) and **15**. The value is the mean of three mice ± standard deviation. (IS) immunosuppression. * Significant differences between control and treated mice for $\alpha = 0.05$.

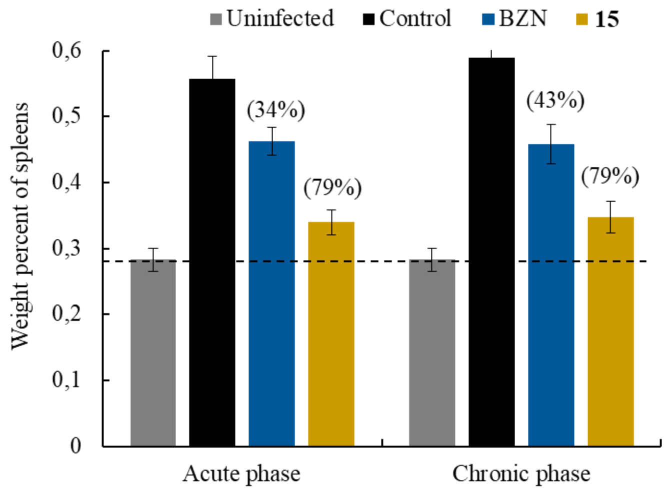

Figure S8. Weight percentage of spleens of mice treated during the acute and chronic infection: control, benznidazole (BZN) and **15**. The value is the mean of three mice ± standard deviation. (IS) immunosuppression. In brackets: reduction of splenomegaly in comparison to the control. Significant differences between control and treated mice for $\alpha = 0.05$.

Table S1. Biochemical clinical parameters for each group of mice: uninfected, control, benznidazole and **15**.

		**Kidney marker profile**		**Heart marker profile**		**Liver marker profile**			
		**Urea (mg/dL)**	**Uric acid (mg/dL)**	**CK-MB (U/L)**	**LDH (U/L)**	**AST/GOT (U/L)**	**ALT/GPT (U/L)**	**Total bilirubin (mg/dL)**	**ALP (U/L)**
**Uninfected mice (n = 15)**		35 [32–40]	4.5 [4.0 –5.1]	372 [150–630]	3180 [2505–3851]	153 [132–177]	55 [46–62]	0.28 [0.22 –0.31]	169 [141–192]
**Treatment in acute phase**	16th dpi (Control) (n = 3)	31	4.3	535	3275	167	60	0.23	180
	16th dpi and BZN (2 days after treatment) (n = 3)	---	----	=	=	++++	++++	++	++
	16th dpi and **15** (2 days after treatment) (n = 3)	-	--	--	=	++	++	+	=
	Necropsy day of mice (Control) (n = 3)	34	4.0	496	2761	179	49	0.21	161
	Necropsy day of mice and BZN (n = 3)	---	=	=	=	++++	=	+	=
	Necropsy day of mice and **15** (n = 3)	=	=	=	=	+	+	+	=
**Treatment in chronic pha**	81th dpi (Control) (n = 3)	45	5.7	751	5951	260	57	0.25	167
	81th dpi and BZN (2 days after	---	----	--	---	++	++	+++	++++

	treatment) (n = 3)								
	81th dpi and **15** (2 days after treatment) (n = 3)	--	---	-	=	+	+	++	=
	Necropsy day of mice (Control) (n = 3)	37	4.8	538	6679	286	64	0.20	149
	Necropsy day of mice and BZN (n = 3)	---	--	=	--	+	=	++	++
	Necropsy day of mice and **15** (n = 3)	-	-	=	=	=	=	+	=

ALP, Alkaline phosphatase; ALT/GPT, alanine aminotransferase; AST/GOT, aspartate aminotransferase; CK-MB, creatine kinase-muscle/brain; LDH, lactate dehydrogenase; BZN, benznidazole; dpi, day post-infection.

Key: =, variation ≤ 10%; +/-, 10-20% increase/decrease over the range; ++/--, 20-30% increase/decrease over the range; +++/---, 30-40% increase/decrease over the range; ++++/----, > 40% increase/decrease over the range.
